# The complete genome sequence of *Kineothrix* sp. MB12-C1, isolated from the feces of black soldier fly larvae

**DOI:** 10.1128/mra.01005-23

**Published:** 2023-12-19

**Authors:** Wenjie Guo, Wanling Qiu, Junyu Zhu, Chao-Jen Shih, Yen-Chi Wu, Shu-Jung Lai, Jiahui Zhou, Zetao Zhu, Yi-Ting You, Yin Li, Sheng-Chung Chen

**Affiliations:** 1 College of Environment and Resources, Fujian Normal University, Fuzhou, Fujian, China; 2 College of Carbon Neutral and Modern Industry, Fujian Normal University, Fuzhou, Fujian, China; 3 School of Resources and Chemical Engineering, Sanming University, Sanming, Fujian, China; 4 Fujian Provincial Key Laboratory of Resources and Environmental Monitoring and Sustainable Management and Utilization, Sanming University, Sanming, Fujian, China; 5 College of Environment and Safety Engineering, Fuzhou University, Fuzhou, Fujian, China; 6 Bioresource Collection and Research Center, Food Industry Research and Development Institute, Hsinchu, Taiwan, China; 7 Graduate Institute of Biomedical Sciences, China Medical University, Taichung City, Taiwan, China; 8 Research Center for Cancer Biology, China Medical University, Taichung City, Taiwan, China; 9 Department of Life Sciences, National Chung Hsing University, Taichung City, Taiwan, China; University of Maryland School of Medicine, Baltimore, Maryland, USA

**Keywords:** black soldier fly, *Kineothrix*, gut microbiota

## Abstract

Here, we present the complete genome sequence of *Kineothrix* sp. MB12-C1 (= BCRC 81406), isolated from the feces of black soldier fly (*Hermetia illucens*) larvae. The genome of strain MB12-C1 was chosen for further species classification and comparative genomic analysis.

## ANNOUNCEMENT

Strain MB12-C1 was isolated from the feces of black soldier fly (*Hermetia illucens*) larvae. The fly eggs were obtained from a shop in Taobao in June 2020 and raised at our school. The larvae were fed wheat flakes and kept indoors in a plastic container at room temperature (~25°C) (1). The fecal sample was collected and inoculated into Methanogen Broth with tungsten (W) (MB/W) media (containing 3% NaCl) as described in previous research (2, 3) on 30 May 2021. After incubation at room temperature for 1 month, the rolling tube technique (4), the Hungate method for the isolation of strict anaerobes, was applied to purify strain MB12-C1. The 16S rRNA gene clone sequencing was employed with 8F (5′-AGAGTTTGATCCTGGCTCAG-3′) and 1492RU (5′-TTTTAATTAAGGTTACCTTGTTACGACTT-3′) primers (5). The purity of the culture was verified by observation of morphology and genome sequencing. The obtained 16S rRNA gene sequence shared the highest similarity of 98.04% to the valid strain, *Kineothrix alysoides* KNHs209^T^ (6), after Standard Nucleotide BLAST (7, 8) analysis. The genome of strain MB12-C1 was chosen for sequencing, specifically for species delineation and conducting comparative genomic analysis.

The strain MB12-C1 has been deposited in the Bioresource Collection and Research Center (Taiwan) as strain BCRC 81406. It was grown in 1 L of anaerobic MB/W media with 3% NaCl and incubated at 30°C. The genomic DNA of strain MB12-C1 was isolated using the NucleoBond HMW DNA kit (Macherey-Nagel) according to the manufacturer’s instructions. The whole-genome shotgun sequencing was performed using the DNBSEQ-T7 platform (MGI Tech Co., Ltd.) and MinION sequencer (Oxford Nanopore Technology) at Sangon Biotech (Shanghai) Co., Ltd.

For the DNBSEQ-T7 platform, sheared genomic DNA fragments of approximately 300 bp were utilized to prepare a 150-bp paired-end DNA library. The library preparation was performed using the Hieff NGS MaxUp II DNA Library Prep Kit for Illumina [Yeasen Biotechnology (Shanghai) Co., Ltd.]. The constructed library was sequenced using the MGISEQ-2000RS High-throughput Sequencing Set (PE150 format), and 17,096,082 reads were generated and trimmed using Trimmomatic v0.36 (9). For the MinION sequencer, sheared DNA fragments were end-repaired, 3′ adenylated, and then ligated to adaptors pre-loaded with motor proteins. The product was purified using Agencourt AMPure XP Beads (Beckman, A63881). Finally, fragments larger than 1 kb were screened for single-molecule nanopore DNA sequencing using the Multiplex Ligation Sequencing Kit (SQK-MLK111.96-XL) on the MinION Flow Cell (ONT, R9.4.1). Basecalling was performed using Guppy version 6.5.6 with a default model (ONT). A total of 86,994 reads were obtained, followed by trimming using Porechop v0.2.4 (10), and filtering using NanoFilt v2.8.0 (11). DNBSEQ-T7 and MinION reads were hybrid *de novo* assembled using Canu v2.2 (12). The assembly *N*_50_ value is 3,935,490 bp. The hybrid sequencing protocol generated ~1,319×mean coverage of the genome. Gene predictions and annotations were performed using NCBI Prokaryotic Genome Annotation Pipeline v6.5 (13).

The assembly generated a single large contig of 3,935,490 bp with 40.8% GC content. It was circularized by aligning and removing overlapping sequences at one end without rotation and predicted 3,636 genes. Default parameters were applied for all bioinformatic analyses.

## Data Availability

The genome sequence of strain MB12-C1 has been deposited in GenBank under accession number CP132957. The version of the genome described in this paper is the first version. The BioProject and BioSample accession numbers are PRJNA1005784 and SAMN36999277, respectively. Raw reads obtained from MinION and DNBSEQ-T7 were deposited in the Sequence Read Archive (SRA) under accession numbers SRR26418082 and SRR26418083, respectively.
